# Decoding the peripheral transcriptomic and meta-genomic response to music in autism spectrum disorder via saliva-based RNA sequencing

**DOI:** 10.3389/fmolb.2025.1696704

**Published:** 2025-11-24

**Authors:** Andrea Cavenaghi, Nour El Zahraa Mallah, Laura Navarro, Federico Martinón-Torres, Alberto Gómez-Carballa, Antonio Salas

**Affiliations:** 1 Unidade de Xenética, Instituto de Ciencias Forenses, Facultade de Medicina, Universidade de Santiago de Compostela, and Genética de Poblaciones en Biomedicina (GenPoB) Research Group, Instituto de Investigación Sanitaria (IDIS), Hospital Clínico Universitario de Santiago (SERGAS), Santiago de Compostela, Spain; 2 Genetics, Vaccines and Infections Research Group (GenViP), Instituto de Investigación Sanitaria de Santiago, Universidade de Santiago de Compostela, Santiago de Compostela, Spain; 3 Centro de Investigación Biomédica en Red de Enfermedades Respiratorias (CIBER-ES), Madrid, Spain; 4 Translational Pediatrics and Infectious Diseases, Department of Pediatrics, Hospital Clínico Universitario de Santiago de Compostela, Santiago de Compostela, Spain

**Keywords:** ASD, microbiome, meta-genomics, transcriptomics, gene expression, RNA-seq, sensogenomics

## Abstract

**Introduction:**

Behavioral interventions for autism spectrum disorder show variable outcomes, highlighting the need for complementary therapies. Music-based interventions are promising, yet their molecular mechanisms remain unclear. Saliva-based RNA sequencing (RNA-seq) provides a non-invasive framework to monitor neuroimmune and metabolic dynamics, but its application in autism remains underexplored.

**Methods:**

We explored the buccal transcriptional effects of music exposure in five individuals with autism (8–37 years; 60% female). To overcome saliva-specific limitations, we combined Poly-A selection and Human-Enriched protocols preparation methods to enhance human transcript detection and reproducibility while capturing microbial signals.

**Results:**

Individually, each dataset revealed a few differentially expressed genes, but integrated analysis improved biological resolution. Consistently modulated genes included *HERC6*, *TSPAN5*, and *REM2*, involved in neurodevelopmental and immune functions. Enrichment analyses highlighted pathways associated with immune regulation, oxidative phosphorylation, and epithelial differentiation, hallmarks of autism, such as immune dysregulation and mitochondrial dysfunction. Co-expression network analysis identified modules correlated with music exposure. The *AKNA* module, previously linked to autism, was downregulated and enriched for Ras-related GTPase and immune pathways, suggesting modulation of intracellular signaling and inflammation. Conversely, upregulation of the *UBE2D3* module indicated activation of endoplasmic reticulum stress responses, a contributor to autism. Exploratory metagenomics identified 15 microbial species responsive to music exposure, including *Acidipropionibacterium acidipropionici* and *Propionibacterium freudenreichii*, producers of propionic acid, a metabolite associated with autism-like behaviors and neuroinflammation.

**Conclusion:**

Saliva-based RNA-seq can stably capture transcriptomic and microbial responses to behavioral stimuli. Music exposure modulates neuroimmune pathways relevant to autism, supporting the biological plausibility of music therapy and demonstrating saliva-based RNA-seq as a viable, non-invasive tool for monitoring intervention outcomes.

## Introduction

Autism Spectrum Disorder (ASD) is a neurodevelopmental condition involving deficits in communication, behavior, and motor skills (World Health Organization, 2021; Kamp-Becker, 2024; American Psychiatric Association, 2022). Symptoms typically emerge between 18 and 24 months, and diagnosis is based on persistent social interaction difficulties, communication challenges, and repetitive behaviors (Zeidan et al., 2022). ASD affects about 1.01% of U.S. children and 0.73% in Europe, with a male-to-female ratio of 4.3. This disparity may be partially due to underdiagnosis of females, who often exhibit subtler symptoms and camouflaging behaviors, suggesting that females may need a higher genetic or environmental threshold to express ASD traits (Volkmar et al., 2014). However, when diagnosed, females often present with more severe symptoms and intellectual disability (Delobel-Ayoub et al., 2020). ASD spans a wide spectrum, from severe impairments to mild social differences (Volkmar et al., 1989), and some individuals also show developmental regression, particularly in language and social skills (Ukkola-Vuoti et al., 2013). Educational, occupational, and social difficulties are frequent. However, others can exhibit exceptional abilities, such as enhanced pitch recognition or visual processing (Samson et al., 2012; Muth et al., 2014; Mazurek et al., 2017; Remington and Fairnie, 2017). Comorbid conditions are common, including intellectual disability (∼31%), sleep disturbances, seizures, anxiety, Attention-Deficit/Hyperactivity Disorder (ADHD), Obsessive-Compulsive Disorder (OCD), and mood disorders (Kielinen et al., 2004; Soke et al., 2018). Needs vary; some people live independently, while others require lifelong support (Seltzer et al., 2004).

ASD’s causes are multifactorial, involving genetic and environmental components. Brain abnormalities such as cerebellar malformations and early overgrowth are being studied as potential causal factors (Hazlett et al., 2017; Stoodley et al., 2017). Genetic research implicates genes involved in brain development and synaptic function (Zoghbi, 2003; McDougle et al., 2005; Voineagu et al., 2011). Environmental risk factors include prenatal exposure to valproic acid or thalidomide, older parental age, low birth weight, and cesarean delivery (Bjørk et al., 2018). Maternal inflammation during pregnancy is another risk factor for ASD. Cytokines like IL-6 and IL-17a, elevated during infections, may disrupt fetal brain development and promote ASD traits (Jash and Sharma, 2021). Postnatal immune profiles also differ in ASD individuals, implicating immune dysregulation. By contrast, folic acid supplementation during pregnancy appears to have a protective effect (Bjørk et al., 2018).

Behavioral interventions remain the cornerstone of ASD management and aim to improve communication, social interaction, and adaptive functioning. Common approaches include applied behavior analysis (ABA), social skills training, and speech or occupational therapy. While these interventions are effective for many individuals, their outcomes can vary and often require long-term, intensive engagement (Lord et al., 2020; Sandbank et al., 2020). Consequently, complementary approaches that enhance motivation, emotional engagement, and social responsiveness have gained attention. In this context, music therapy may represent a particularly promising avenue, as it engages widespread brain regions and influences emotion, cognition, and motor skills (Zatorre and McGill, 2005). It stimulates dopamine release, affects autonomic responses, and promotes neuroplasticity. Its effects vary by age, sex, culture, and musical training (Zatorre and McGill, 2005; Kanduri et al., 2015). Music therapy is used in diverse clinical contexts such as addiction, Post-Traumatic Stress Disorder (PTSD), dementia, and brain injury (The American Music Therapy Association, 2014). In ASD, music is promising due to the usually higher auditory sensitivity and pitch memory of the patients (Remington and Fairnie, 2017). While auditory sensitivity can lead to overload in ASD patients, it also allows deep engagement with music’s predictability and structure (Goris et al., 2020). Active and passive music therapy can support not only emotional regulation and social communication but also improve emotional processing, motivation, attention, and interaction (Cook et al., 2019; Navarro et al., 2025a; Navarro et al., 2025b; Salas et al., 2025). Structured programs, in turn, strengthen parent-child relationships while reducing symptom severity (Lense et al., 2020). Music stimulation has been shown to increase oxytocin and vasopressin, neuropeptides involved in empathy and bonding. Oxytocin enhances social cue recognition and has been genetically linked to ASD (Domes et al., 2007). Compared to speech therapy or medication, music therapy often yields greater improvements in life quality (Israel et al., 2008). Neuroimaging studies have shown that music therapy enhances brain connectivity, especially between auditory and motor regions (Sharda et al., 2018). However, most research studies focused on children and lacked standardization. Given individual variability in music sensitivity, personalized approaches are essential. In this context, the emerging field of sensogenomics investigates how sensory stimuli like music influence gene expression and brain function, offering new insights into ASD.

Many children with ASD exhibit significant fear and avoidance of medical procedures, particularly those involving needles (Sukhodolsky et al., 2008; Wolff and Symons, 2013) and, therefore, alternative less invasive approaches are under investigation. Though exposure therapy may help reduce stress related to blood draws, its application is limited by safety and logistical constraints (Meindl et al., 2019). In this context, saliva presents a non-invasive, easily collectable alternative for biomarker discovery, supplying DNA, RNA, and proteins for molecular analysis, including identification of polymorphisms linked to ASD. Saliva is increasingly recognized as a valuable non-invasive biofluid for biomedical research and biomarker discovery. In neurological conditions, saliva is being explored for monitoring brain health (Farah et al., 2018; Engeland et al., 2019). Parasympathetic and sympathetic stimulation of salivary glands triggers acetylcholine and noradrenaline release to the buccal cavity, respectively, promoting protein secretion; these neurotransmitters have been detected in rodent salivary glands (Murai et al., 1998; Nakamura et al., 2004; Proctor and Carpenter, 2007). The nervous regulation of the salivary glands supports the concept of a bidirectional oral-brain axis, where local inflammatory stages may influence each other (Sansores-Espana et al., 2021). In fact, different hormonal biomarkers, such as cortisol and oxytocin, have been studying in saliva from ASD patients (Majewska et al., 2014; Bakker-Huvenaars et al., 2020). Studies in different tissues, including saliva, have also shown that oxytocin levels are lower in ASD children (John and Jaeggi, 2021). Several studies have tried to “treat” ASD social impairments with oxytocin some of them showed improvements (Yatawara et al., 2016; Parker et al., 2017), while others showed no differences between the oxytocin treatment and the placebo treatment (Anagnostou et al., 2012; Munesue et al., 2016). In the context of ASD, small RNA molecules such as microRNAs (miRNAs), piRNAs, and snoRNAs are involved in neurodevelopment and synaptic regulation. In a 2018 study, 32 RNA markers in saliva distinguished ASD from controls with 85% accuracy. Changes in miRNAs like *miR-146b-5p* and *piR-6463* were found diagnostically relevant (Hicks et al., 2018). *miR-141-3p* expression was found to be negatively correlated with oral bacteria of the genus *Tannerella* in ASD children’s saliva, suggesting that dysregulation of miRNAs and the oral microbiome dysbiosis may be linked to cognitive impairments (Frey et al., 2018; Ragusa et al., 2020; Kamble et al., 2024). Indeed, 70% of ASD children have gastrointestinal symptoms and are often associated with greater symptoms severity (Benach et al., 2012; Lefter et al., 2019). Numerous investigations have reported different microbial profiles in individuals with ASD compared to neurotypical controls, suggesting potential effects on ASD-related pathways (Iglesias-Vazquez et al., 2020; Peralta-Marzal et al., 2024). Although specific microbial differences associated with ASD remain inconsistent across studies, accumulating evidence supports a crucial role of the bidirectional microbiota gut–brain axis in ASD and other neurodevelopmental disorders (Cryan et al., 2019; Peralta-Marzal et al., 2021). Microbial metabolites can cross the intestinal barrier and enter systemic circulation, where they may exert effects on distant organs and tissues, such as the brain (Popoff, 2011). It has been hypothesized that some species of *Clostridium* can release neurotoxins that could influence the ASD pathology. For instance, *Clostridium difficile* is an emerging pathogen associated with antibiotic-related diarrhea and increased gut permeability. Individuals with ASD are frequently exposed to intensive antibiotic regimens, heightening their susceptibility to *C. difficile* infection (Kuhn et al., 2012). Studies in mouse models indicate that *C. difficile* can alter p-cresol levels and dopamine-β-hydroxylase activity, affecting dopaminergic signaling in the brain, suggesting a potential link between *C. difficile* and neurodevelopmental alterations in ASD (Kakabadze et al., 2022). However, other studies have reported contrasting findings, showing no significant association between ASD and *C. difficile* (Khalil et al., 2021).

Despite its advantages, saliva-based diagnostics face technical challenges. Only about 20% of RNA sequences align with the human genome, while 30% match known oral microbes, leaving 50% unclassified, likely representing fungi, viruses, or degraded fragments (Rylander-Rudqvist et al., 2006; Spielmann et al., 2012; Frey et al., 2018; Kamble et al., 2024). Human RNA is easily degraded, and bacteria produce abundant RNA. In datasets like RNAgE-WT, human RNA comprised just 0.1% of reads, while bacterial RNA exceeded 75% (Gosch et al., 2024). Low analyte concentrations, sample variability, and degradation during handling (e.g., freeze-thaw cycles) further complicate analysis (Gröschl et al., 2001; Christodoulides et al., 2005; Pfaffe et al., 2011).

Exploring music’s effect on gene expression in ASD through saliva offers a promising, non-invasive research avenue. However, it involves two major obstacles: the complexity of human vs. microbial RNA content, and the novelty of assessing transcriptional changes induced by music in a population with neurobiological heterogeneity. Addressing these challenges could advance personalized music-based therapies.

This pilot study evaluated the feasibility of using saliva to analyze human RNA changes before and after music exposure in individuals with ASD. We employed two complementary methods of RNA-seq library preparation, and the results revealed reproducible transcriptomic responses, supporting saliva’s potential as a non-invasive tool for exploring the neurobiological impact of music in ASD. These findings lay the groundwork for future research into personalized, music-based interventions for neurodevelopmental disorders.

## Materials and methods

### Participants and sample collection

This study was conducted as part of the Sensogenomics project (https://sensogenomics.com; Navarro et al., 2021; Gómez-Carballa et al., 2023; Navarro et al., 2023; Gómez-Carballa et al., 2025a; Gómez-Carballa et al., 2025b), which explores the biomolecular and physiological effects of music in diverse population groups. The experimental concert, Sensogenoma22, was performed by Real Filharmonía de Galicia, conducted by Baldur Brönnimann in the Auditorio de Galicia (Santiago de Compostela, Spain). A selected repertoire of 50 min, trying to capture attention and impulse emotions through the variety of timbres, tempos, styles, and tonalities: The Unsewered Question (C. Ives), The Merry Wives of Windsor (O. Nicolai), Slavonic Dances n° 2 and n° 3, op. 46 (A. Dvořák), Oblivion (A. Piazzolla), Hungarian Dance n° 5 (J. Brahms), The Barber of Seville: Overture (G. Rossini), Danzón n° 2 (A. Márquez). To minimize response bias, the musical program was not disclosed in advance. All participants remained seated throughout the performance to eliminate the influence of physical activity. The environment was carefully designed to be comfortable, calm, and non-invasive, thereby reducing stress or other external factors that could affect physiological responses. The audience consisted of individuals from distinct groups, including individuals diagnosed with ASD, healthy control participants, and general spectators. For the present pilot study, a subset of five individuals with ASD was selected. The mean age of the ASD subjects was 23.8 years (8–37 years), and 60% were females. To ensure the reliability and comparability of transcriptomic data, all participants were instructed to abstain from eating, drinking, smoking, chewing gum, or engaging in physical activity for at least 30 min before saliva collection.

Saliva samples were collected immediately before and after the concert, simultaneously for all participants. The collection was performed under the supervision of trained healthcare professionals to guarantee consistency and adherence to the protocol. Each participant used an Oragene RNA saliva collection device (ORE-100, DNA Genotek), which is optimized for the non-invasive collection, stabilization, and transport of high-quality RNA from saliva. Participants were instructed to rub the sponge tips of the device along both sides of their gums, avoiding contact with teeth, until sufficient saliva was absorbed. The sponge tips were then inserted into tubes containing 1 mL of stabilizing solution, which preserves RNA integrity. Samples were subsequently stored at room temperature until processing.

**FIGURE 1:**
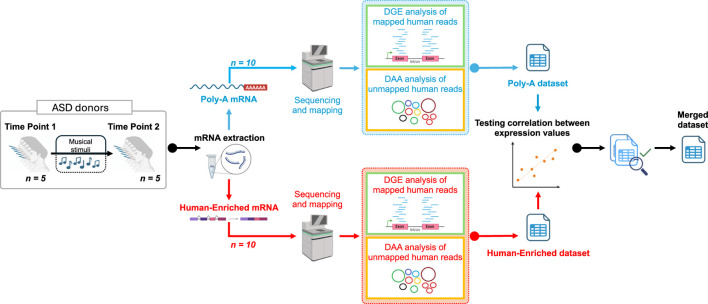
Graphic scheme showing the design of the present work. The figure was built using Biorender resources (https://biorender.com/).

Written informed consent was obtained from the legal guardians or responsible parties of all participating individuals. The study was approved by the Ethics Committee of the Xunta de Galicia (registration code: 2020/021) and conducted in accordance with the principles outlined in the Declaration of Helsinki. Figure 1 shows a scheme of the experimental design used in the present study.

### RNA isolation and RNA-sequencing

RNA was isolated from 500 μL of saliva using the RNeasy Micro Kit (Qiagen), using the protocol provided by the extraction kit as recommended by the Oragene tubes supplier. Samples were subjected to a DNase treatment to remove DNA. Further purification, concentration, and DNAse treatment were performed using the RNA Clean & Concentrator Kit (Zymo). Final RNA concentration was verified with Nanodrop, yielding >50 ng/μL, suitable for downstream applications.

RNA quantity and integrity (RIN) were assessed using the TapeStation 4,200 system (Agilent), and RNA concentration was quantified using the Qubit fluorometer (Thermo Fisher Scientific). Two different Illumina library preparation strategies were employed: i) Illumina RNA Prep with Enrichment (https://emea.illumina.com/products/by-type/sequencing-kits/library-prep-kits/rna-prep-enrichment.html), which uses hybrid capture to enrich for specific RNA targets, including non-coding RNAs, degraded RNA, or low-input samples (hereafter referred to as the Human-Enriched library), and ii) TruSeq Stranded mRNA, which selectively captures polyadenylated mRNA (poly-A selection), primarily targeting coding transcripts and thereby focusing on protein-coding gene expression (hereafter referred to as the Poly-A library). Human-Enriched libraries were prepared using the Illumina RNA Prep with Enrichment (ref. 20040537) workflow, which includes RNA fragmentation, reverse transcription into cDNA, adapter ligation, library amplification, purification, and target enrichment via hybridization capture using the Illumina Exome Panel (ref. 20020183). For the Poly-A library, strand-specific libraries were generated using the TruSeq Stranded mRNA technology (ref. 20020594). Indexing was performed using unique dual indexes with the IDT for Illumina TruSeq RNA UD Indexes kit (ref. 20022371).

Sequencing for both library types was performed on the NovaSeq 6000 System (Illumina) using a paired-end configuration (2 × 100 bp). Each sample generated approximately 30 million paired-end reads when using the Human-Enriched and 60 million paired-end reads with the Poly-A, with an average of 85% of bases achieving a quality score above Q30.

### Transcriptomic data processing and statistical analysis

To assess the quality of the raw reads, *FastQC* (v0.11.9) and *MultiQC* were employed. Following the initial quality assessment, reads were processed using *Trimmomatic* (Bolger et al., 2014) to remove low-quality bases and residual adapter sequences. Reads were further filtered using the sliding window approach, where sequences are scanned from the 5′ to 3′ end and trimmed when the average quality within a defined window falls below a threshold. High-quality reads were aligned to the *Homo sapiens* reference genome (GRCh38/hg38) using *STAR* (Spliced Transcripts Alignment to a Reference) version 2.7.9a (Dobin et al., 2013). Gene-level quantification was performed using the *HTSeq* package (version 2.0.3) (Putri et al., 2022). Data normalization and differential expression (DE) analysis was conducted using the *DESeq2* package (version 1.46.0) in RStudio (v4.4.2) (Love et al., 2014). To evaluate the robustness and reproducibility of the results, two datasets derived from different RNA isolation methods, Human-Enriched and Poly-A selected libraries, were compared. Exploratory data visualization was performed using histograms of read counts, Principal Component Analysis (PCA), hierarchical clustering heatmaps, and volcano plots. These analyses were executed with R packages *ggplot2* (Wickham, 2016), *ComplexHeatmap* (Gu, 2022), and *EnhancedVolcano* (Blighe et al., 2020). *DESeq2* package was used to detect differentially expressed genes (DEGs) after the musical stimuli when compared to the baseline. We include the patient ID in the model as a random effect to account for patient-to-patient differences in the baseline. Genes common to both datasets and displaying statistically significant differential expression (*P*-value <0.05) were examined via Pearson correlation to assess consistency across the two preparation methods. Upon confirming a strong correlation, a merged dataset was constructed by summing the read counts of shared genes. This integration strategy aimed to enhance the sensitivity of the analysis and mitigate false negatives associated with the limitations of each isolation protocol.

Functional enrichment analysis was performed on the merged dataset. First, a differential pathways analysis was performed using the Gene Set Variation Analysis (*GSVA*) R package (Hänzelmann et al., 2013). Gene sets corresponding to Gene Ontology (GO) biological processes were obtained from the Molecular Signatures Database (MSigDB) and used as the reference collection. Differentially regulated pathways (DRPs) were identified using the *limma* package (Ritchie et al., 2015), applying an adjusted *P*-value threshold of <0.05, with TP1 as the reference group. Additionally, we conducted an over-representation analysis (ORA) of GO terms with the *ClusterProfiler* R package (Wu et al., 2021; Yu, 2024), *org.Hs.eg.db* (Carlson, 2023), and *AnnotationDbi* (Pagès et al., 2024). These tools facilitated the identification of pathways significantly enriched among DEGs, providing insight into the biological processes modulated in response to the experimental conditions. For specific analyses, we generated a subset of neurobiological processes by systematically and manually curating the GO (biological processes) database, selecting entries that included neurobiologically related terms. This approach resulted in a total of 483 biological processes (Supplementary Table S1).

We next compared the DEGs found in the merged dataset with those indexed in the Simons Foundation Autism Research Initiative (SFARI) database (Abrahams et al., 2013). This database, developed for the ASD research community, compiles information on genetic variants associated with the etiology of ASD. Genes in the SFARI database are classified into four categories: i) syndromic (genes with mutations strongly linked to ASD and additional clinical features beyond core diagnostic criteria), ii) Level 1 (high-confidence ASD risk genes), iii) Level 2 (strong candidate genes), and iv) Level 3 (genes with moderate evidence based on previous studies). This comparison allowed us to assess the extent to which our findings align with the existing literature on ASD genetics.

### Co-expression modules analysis

To explore gene co-expression patterns potentially associated with musical stimuli in the buccal mucosa of ASD patients, we constructed a signed weighted correlation network using the Weighted Gene Co-expression Network Analysis (*WGCNA*) R package (Langfelder and Horvath, 2008). Gene expression data of the merged dataset were first normalized and corrected for inter-individual variability. To focus on the most informative signals, we retained the top 75% of genes with the highest variance across samples. A soft-thresholding power of 26 was selected based on scale-free topology criteria, yielding a model fitting index above 0.80 for both datasets (Supplementary Figure S1A). We computed the Topological Overlap Matrix (TOM) and corresponding dissimilarities (1–TOM), followed by hierarchical clustering. Modules were defined using a minimum size of 30 genes and merged using a dendrogram cut height of 0.2 (Supplementary Figure S1B). Each resulting module was assigned a unique color identifier. Module eigengenes were correlated with the TP1-TP2 to identify modules significantly associated with musical stimulation, based on gene significance (GS). Within each co-expression module identified, Module Membership (MM) for each gene was calculated as the correlation between each gene’s expression profile and the module eigengene (the first principal component summarizing the expression pattern of all genes within that module). MM thus reflects the degree of intramodular connectivity, indicating how strongly a gene is associated with the overall expression trend of its module. Genes with high MM values were considered central, as they likely play key regulatory roles in the biological processes represented by the module. MM was used to identify hub genes (those with the highest connectivity). Each significant module was labeled according to its hub gene name.

To assess the biological relevance of the significant modules, a clustered over-representation was conducted using the *compareCluster* function from the *clusterProfiler* R package (Wu et al., 2021), which enabled the parallel evaluation of all genes belonging to each significant module, irrespective of their MM. GO biological process terms were used as a reference database. We reduce redundancy in GO terms with the *simplifyEnrichment* R package (Gu and Hubschmann, 2023), using the binary cut method for clustering the similarity matrix of biological terms.

### Exploratory metagenomic analysis of salivary samples

To investigate potential shifts in bacterial composition associated with musical stimulation, we conducted an exploratory metagenomic analysis using reads that did not align to the human reference genome. These unmapped reads were extracted using the STAR aligner with the --*outReadsUnmapped Fastx* parameter. Read quality was assessed with *FastQC* and summarized using *MultiQC*, revealing consistently high base quality scores across all samples. Common metagenomic artifacts, such as per-base sequence content biases, sequence duplication, and adapter contamination, were detected. These issues were addressed using *Trimmomatic*, which efficiently removed adapter sequences and improved overall read quality. These unmapped reads were processed and classified against a bacterial genome database using the *CLARK* metagenomic classifier (v1.3.0.0) (Ounit et al., 2015; Ounit and Lonardi, 2016), which provides a taxonomic profile based on discriminative *k*-mers (short DNA sequences of fixed length *k*) and assigns bacterial labels based on discriminative *k*-mer comparisons against the NCBI/RefSeq bacterial genome database. *CLARK* constructs an index from reference genomes by identifying unique *k*-mers that are specific to each genome. Shared *k*-mers among multiple genomes are excluded to ensure that only genome-specific sequences are used for classification. During the read assignment, *CLARK* quantifies the number of shared discriminative *k*-mers between each read and reference genomes, classifying each read to the genome with the highest match. The analysis was conducted in full mode, which utilizes the complete set of discriminative *k*-mers and outputs detailed metrics, including read counts per taxon and confidence scores (ranging from 0.5 to 1.0). This mode was selected to retain maximum resolution and accuracy in taxonomic assignments.

*DESeq2* package was used to normalize the data and detect differences in the abundance of microorganisms after the musical stimuli when compared to the baseline. We include the patient ID in the model as a random effect to account for patient-to-patient differences in the baseline using the *limma* package.

Graphical visualizations were generated in RStudio (v4.4.2) using the *ggplot2* and *ggwaffle* packages (Rudis and Gandy, 2015). Visual outputs included bar plots of the most abundant bacterial genera and comparative plots of relative and absolute abundances before and after musical exposure.

## Results

### Quality assessment of raw data and comparative evaluation of the mapping from library preparation protocols

Initial quality control assessments revealed that the raw sequencing reads were of very high quality across all samples, with optimal values observed for most quality parameters (data not shown). However, two quality metrics were consistently flagged across all samples: “Per Base Sequence Content” and “Sequence Duplication Levels.” These warnings, though persistent, are not unusual in RNA-seq experiments and are not indicative of poor sample quality. In contrast, the presence of adapter contamination indicated a clear need for improvement. To address this, sequencing adapters and low-quality bases were removed using *Trimmomatic*. A comparative quality assessment between Human-Enriched and Poly-A RNA-seq libraries in both pre-processed and trimmed datasets revealed several differences across different sequencing metrics, reflecting intrinsic characteristics of the two library preparation strategies. The most remarkable difference between the two methods is related to the duplication rates. Human-Enriched library displayed higher levels of duplication, with a mean rate of 84.04% in the pre-processed data and 85.42% in post-trimming data (Supplementary Table S2). In contrast, Poly-A libraries exhibited markedly lower duplication, with an average of 61.88% pre-trimming and a moderate increase to 67.89% after trimming. GC content also differed between the two library types. Human-Enriched library maintained a consistently higher GC percentage, averaging 52%–53% across both pre-trimmed and trimmed data, but, in contrast, Poly-A library exhibited a lower GC content, ranging from 42.95% in the pre-processed data to 44.1% post-trimming (Supplementary Table S2). Finally, a substantial reduction in failed reads was observed following trimming in both library types.

**FIGURE 2:**
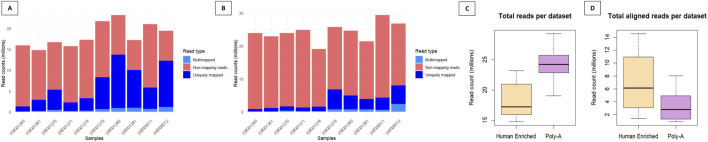
Alignment metrics and number or reads per sample across datasets. Multimapping, non-mapping and uniquely mapping reads illustrating the alignment performance of the Human-Enriched dataset **(A)** and the Poly-A dataset **(B)** against the human genome (hg38). **(C)** Boxplots comparing the total number of reads in the Human-Enriched dataset and the Poly-A dataset. **(D)** Boxplots comparing the total number of reads aligned to the human genome (hg38) in the Human-Enriched dataset and the Poly-A dataset.

To evaluate mapping efficiency and alignment quality, the output log files from STAR were aggregated and analyzed using *MultiQC*. Overall, poor alignment performance was observed in both the Human-Enriched and Poly-A datasets, characterized by a high proportion of reads that failed to map to the human reference genome (Figures 2A,B). This result was not unexpected, given the RNA source, saliva, which contains a substantial amount of microbial and other non-human genetic material. These unmapped reads were subsequently analyzed through a dedicated metagenomic pipeline to investigate the composition and modulation of the oral microbiome in response to musical stimulation. A comparative assessment of the two datasets revealed notable differences attributable to their respective library preparation methods. The Poly-A dataset generally yielded a higher total number of reads, both mapped and unmapped, as shown in Figure 2C (see also Supplementary Table S3). However, despite this greater sequencing depth, a smaller proportion of reads in the Poly-A dataset successfully aligned to human genes compared to the Human-Enriched dataset (Figure 2D).

Further analysis showed that the Poly-A dataset achieved broader transcriptomic coverage, detecting approximately 20,390 genes. However, this broader coverage came at the cost of lower average read counts per gene. In contrast, the Human-Enriched dataset, as expected, showed a more focused mapping pattern: while fewer genes were detected (approximately 17,730), the number of reads mapping to each gene was higher, indicating increased sequencing depth per gene.

### Differential gene expression analysis

A differential expression analysis was carried out to detect DEGs after the musical stimuli in the two salivary datasets separately. To improve statistical power and reduce noise, genes with fewer than 10 counts in at least five samples were filtered out. After applying this filtering criterion, 9,455 genes were retained in the Human-Enriched dataset, while only 6,791 genes were retained in the Poly-A dataset. The lower number of retained genes in the Poly-A dataset likely reflects its lower average read count per gene and sample.

**FIGURE 3:**
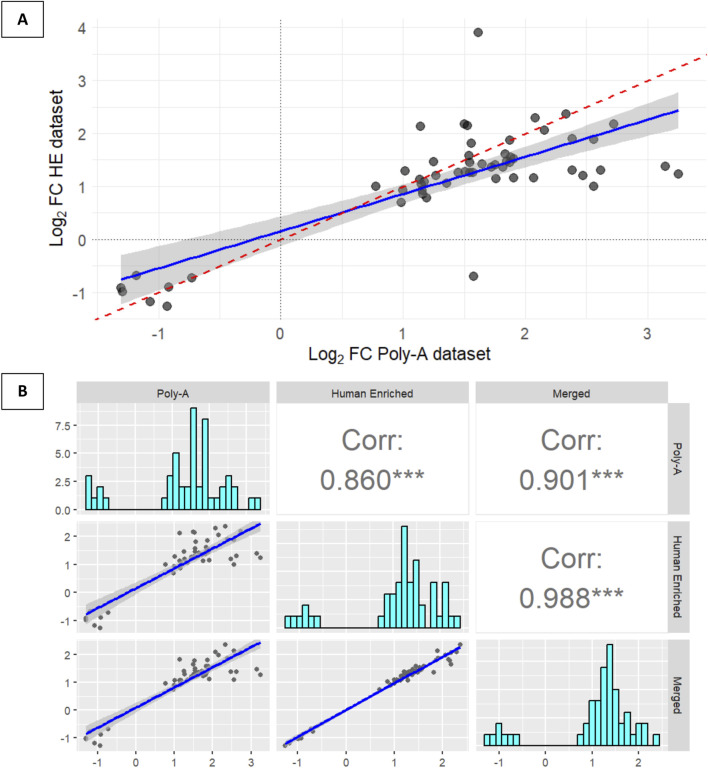
Correlation analyses between significant expression changes (TP1 vs. TP2) from different datasets. **(A)** Plot showing the correlation of Log_2_FC values from 57 differentially expressed genes (*P*-value <0.05) common between the Human-Enriched and Poly-A datasets (correlation = 0.77; 95% confidence interval). **(B)** Correlation between Log_2_FC values from 50 differentially expressed genes (*P*-value <0.05) shared between the merged dataset and the 57 common genes previously identified from the analysis of Human-Enriched and Poly-A datasets.

When considering genes with nominal *P*-values <0.05, the Human-Enriched and Poly-A datasets yielded 720 and 442 genes, respectively. Of these, 57 genes were shared between the two datasets. Among these common genes, 55 were protein-coding, one was a long non-coding RNA, and one was a transcribed unprocessed pseudogene. To assess the concordance in expression trends, we compared Log_2_FC values for all DEGs identified in both datasets. The Log_2_FC values of all DEGs identified in either dataset (*n* = 738), regardless of whether they were detected in one or both, showed strong agreement in the direction of gene expression changes (i.e., genes upregulated in Poly-A were also found to be upregulated in the Human-Enriched subset, and *vice versa*). This concordance was supported by high-performance metrics, including an accuracy of 0.85, precision of 0.87, recall of 0.90, an F1-score of 0.88, and a Cohen’s kappa coefficient of 0.68 (Supplementary Table S4). In addition, when examining only the shared DEGs detected in the two datasets (*n* = 57), these metrics improved substantially, with an accuracy of 0.98, precision of 0.98, recall of 1.00, an F1-score of 0.99, and a Cohen’s kappa coefficient of 0.92 (Supplementary Table S4). Finally, the concordance in Log_2_FC between the 57 shared DEGs revealed a significant correlation between the Human-Enriched and Poly-A datasets (*R* = 0.77; Figure 3A). These results suggest that, despite technical differences and inherent biases between the two library preparation methods, the integration strategy used to combine both datasets successfully captured overlapping transcriptional responses to the musical stimulus.

### Integration through a merged dataset and cross-dataset validation of expression trends

To further optimize the analysis, a merged dataset was created by summing the read counts of shared genes across samples that were present in both datasets. This integration approach aimed to combine the broader gene coverage offered by the Poly-A dataset with the higher per-gene sequencing depth of the Human-Enriched dataset. Each dataset was filtered individually using the same criteria (genes with ≥10 counts in at least five samples). Afterward, counts from both datasets were merged by summing their values, followed by normalization and differential expression analysis using *DESeq2* (Supplementary Table S5). To further confirm the consistency of expression patterns in the two datasets, genes with *P*-values below 0.05 in the merged dataset (*n* = 433) were intersected with the 57 genes previously found to be shared between the Human-Enriched and Poly-A datasets. A total of 50 genes were common to all three analyses. The Log_2_FC values for these genes were again compared across datasets. The Pearson correlation coefficient was 0.86 when comparing the Human-Enriched and Poly-A libraries, and exceeded 0.90 when each was compared individually with the merged dataset (Human-Enriched vs. Merged: 0.988; Poly-A vs. Merged: 0.901). All three comparisons were highly statistically significant (Figure 3B). These correlation values indicate strong consistency in expression patterns and further support the validity of the merging approach.

**FIGURE 4:**
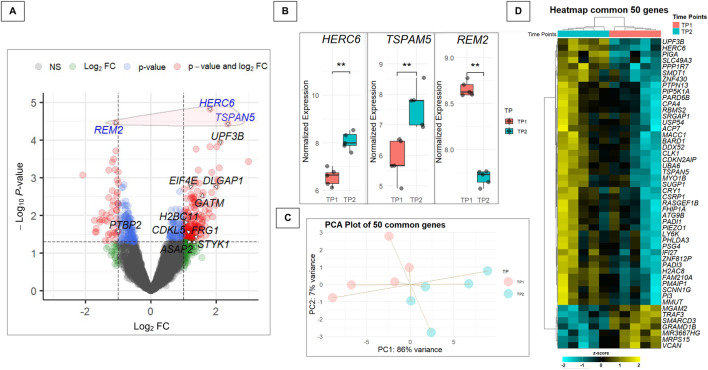
Analysis of DEGs and their relevance to autism-related pathways. **(A)** Volcano plot of the merged dataset, highlighting genes listed in the SFARI database and encircling in red the three genes with adjusted *P*-value <0.1 that are not included in the SFARI database. The plot shows the Log_2_FC on the x-axis and the -log_10_ *P*-value on the y-axis. Genes with a *P*-value <0.05 and a |Log_2_FC| > 0.5 are colored in red, genes with a *P*-value <0.05 but a |Log_2_FC| < 0.5 in light blue, genes with an *P*-value >0.05 but a |Log_2_FC| > 0.5 in green and non-significant genes in dark grey. **(B)** Boxplot showing the expression distribution before (TP1) and after (TP2) the musical stimuli of the 3 DEGs listed in the SFARI database in the merged dataset (**P-value = 0.0079). **(C)** Principal Component Analysis (PCA) of the 50 differentially expressed genes (*P*-value <0.05) shared between the merged dataset and the 57 genes previously found in common between the Human-Enriched and Poly-A datasets (*P*-value <0.05), illustrating sample distribution based on gene expression profiles. **(D)** Heatmap displaying the expression patterns of the 50 genes from the intersection between the differentially expressed genes (*P-value* <0.05) in the merged dataset and the 57 genes previously found in common between the Human-Enriched and the Poly-A datasets (*P*-value <0.05).

The merged dataset revealed three DEGs after musical stimuli when compared to baseline, with adjusted *P*-values <0.1 (Figure 4A). Other genes previously associated to ASD were also significantly affected by the musical stimuli. Two genes, *HERC6* and *TSPAN5*, were upregulated, while *REM2* was downregulated (Figure 4B). Gene expression profiles of the 50 common genes could perfectly separate TP1 from TP2 in both the PCA (Figure 4C) and the heatmap analysis (Figure 4D).

### Functional interpretation and pathway exploration

Although the number of statistically significant DEGs identified in this study was limited, the high consistency observed across multiple datasets and analytical approaches strongly supports the hypothesis that music exposure can elicit measurable transcriptional changes in the buccal mucosa of individuals with ASD. To further explore the potential biological relevance of these changes, we performed pathway-level analyses using both GSVA and ORA. These complementary methods allowed us to move beyond single-gene effects to identify broader biological pathways and molecular functions modulated by musical intervention.

**FIGURE 5:**
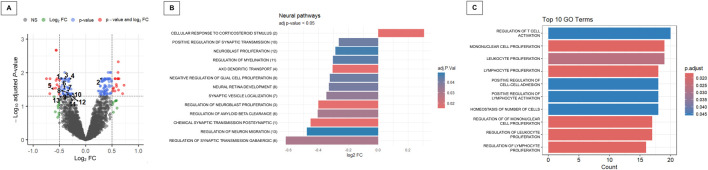
Comprehensive functional enrichment analysis of the merged dataset across multiple approaches. **(A)** Volcano plot of differential pathways analysis identified via Gene Set Variation Analysis (GSVA); The plot shows the Log_2_FC on the x-axis and the -log_10_ adjusted *P*-value on the y-axis. Genes with an adjusted *P*-value <0.05 and a |Log_2_FC| > 0.5 are colored in red, genes with an adjusted *P*-value <0.05 but a |Log_2_FC| < 0.5 in light blue, genes with an adjusted *P*-value >0.05 but a |Log_2_FC| > 0.5 in green and non-significant genes in dark grey. The numbers in the plot correspond to GSVA-enriched (adjusted *P*-value <0.05) neuronal biological functions (see Supplementary Tables S1, S5). **(B)** Barplot of significantly GSVA-enriched pathways related specifically to neuronal biological functions (adjusted *P*-value <0.05); see Supplementary Tables S1, S5 for details. **(C)** Barplot summarizing the top 10 significantly enriched pathways (adjusted *P*-value <0.05) identified through Over-Representation Analysis (ORA).

The differential pathway analysis revealed 137 DRPs in ASD patients (adjusted *P*-value <0.05), of which 58% (*n* = 79) were upregulated and 42% (*n* = 58) were downregulated following music exposure (Figure 5A; Supplementary Table S6). Among the most significantly affected pathways (|Log_2_FC| > 0.5 and adjusted *P*-value <0.01), we identified biological processes such as aorta development, actin filament severing, endoplasmic reticulum to cytosol transport, and skeletal muscle tissue regeneration. Notably, 13 of the 137 DRPs were associated with neurobiological functions (Figure 5B; Supplementary Table S6).

Interestingly, in contrast to the global trend toward upregulation, neurobiological pathways showed a predominance of downregulation: 12 out of 13 exhibited negative Log_2_FC values post-stimulation, suggesting reduced activity after music exposure. These included key processes such as chemical synaptic transmission, postsynaptic (Log_2_FC = −0.45, adjusted *P*-value = 0.015), regulation of neuroblast proliferation (Log_2_FC = −0.40, adjusted *P*-value = 0.015), and axo-dendritic transport (Log_2_FC = −0.31, adjusted *P*-value = 0.018), pointing toward a potential dampening of excitatory synaptic signaling and neurodevelopmental activity. The only upregulated neurobiological pathway (Log_2_FC = 0.31, adjusted *P*-value = 0.015) was the cellular response to corticosteroid stimulus. Additionally, regulation of GABAergic synaptic transmission was notably downregulated (Log_2_FC = −0.62, adjusted *P*-value = 0.030). Other affected pathways involved in synaptic plasticity, neurodevelopment, and neurodegeneration, such as regulation of amyloid-beta clearance, synaptic vesicle localization, and neuron migration, were also significantly suppressed.

Functional analysis using the ORA approach identified 19 differentially regulated pathways (Figure 5C; Supplementary Table S7), many of which were associated with immune-related processes. These pathways included those involved in immune T-cell proliferation and activation, migration and adhesion, as well as the maintenance of immune homeostasis.

Comparison between both unsupervised (GSVA) and ORA approaches revealed only a common DRP, namely “calcium ion import across plasma membrane”, which was downregulated after the musical stimuli (Log_2_FC = −0.74, adjusted *P*-value = 0.015). However, an additional pathway related to T-cell homeostasis also emerged from the GSVA results (“regulation of t cell differentiation in thymus”; Log_2_FC = −0.43, adjusted *P*-value = 0.04), reinforcing the involvement of T-cell-related pathways in the response to music.

### Co-expression modules analysis in response to musical stimulation

**FIGURE 6:**
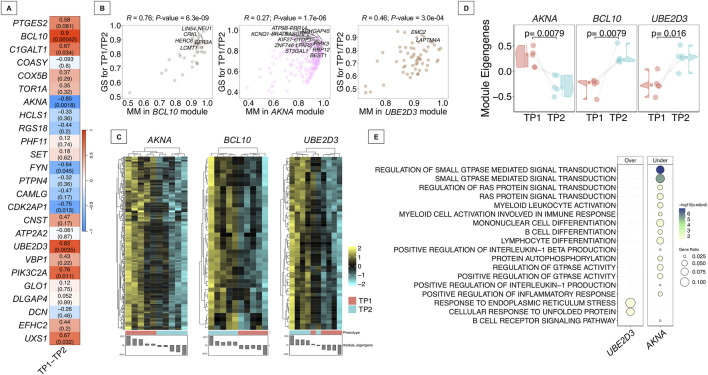
Co-expression analysis in ASD donors. **(A)** Heatmap of correlation values between modules of co-expressed genes detected and the musical stimuli (TP1-TP2). The upper value corresponds to the individual correlation value of each of the modules. *P*-values of these correlations are displayed in brackets. **(B)** Comparison between MM (module membership) and musical stimuli correlation of genes from the most significant module detected (adjusted *P*-value <0.05). Only names from genes showing an MM > 0.9 and a correlation with the musical stimuli >0.9 are displayed. **(C)** Heatmap of gene expression profiles from the modules most significantly correlated with the musical stimuli (adjusted *P*-value <0.05). **(D)** Raincloud plots of differences in individual samples’ eigengenes between TP1 and TP2 from modules showing statistical significance (adjusted *P*-value <0.05). **(E)** Biological processes detected from most statistically significant modules (adjusted *P*-value <0.05). Modules with significant pathways are named as the hub gene names.

*WGCNA* analysis was conducted on a total of 4,667 genes from the merged dataset, after filtering out less variable genes. The analysis detected 28 modules of co-expressed genes, and among them, eight modules were significantly correlated with the musical stimuli. Multiple test correction was applied (FDR < 0.05), resulting in three statistically significant correlated modules (|*R*| > 0.8); Supplementary Table S8. Two of these, bisque4 (hub gene: *BCL10*) and saddlebrown (*UBE2D3*), exhibited strong positive correlation, while one module, plum1 (*AKNA*), showed a strong negative correlation with the music stimuli (Figure 6A). Other modules, such as darkolivegreen (*CDK2AP1*) and steelblue (*PIK3C2A*), also demonstrated notable correlation (*r* = −0.75 and *R* = 0.76, respectively), although they did not retain statistical significance after correction (adjusted *P*-value = 0.065 for both). Equally, other additional modules, including ivory (*C1GALT1*), thistle1 (*UXS1*), and lightgreen (*FYN*) exhibited moderate correlations (*r* ranging from 0.64 to 0.67), but did not reach significance following adjustment (Supplementary Table S8). Correlation between module membership (MM) and gene-level correlation with the musical stimuli of the three significant modules demonstrated a robust and statistically significant association, supporting the idea that core genes within these modules are functionally aligned with the musical response (Figure 6B). The most substantial association was observed in the *BCL10* module (*r* = 0.76; *P*-value = 6.3e-09). Heatmap analysis of gene expression patterns from genes included in each of the significantly associated modules evidenced their overall over-regulation (*BCL10* and *UBE2D3* modules) and under-regulation (*AKNA* module) after listening to music (Figure 6C). Eigengene values of individual samples also align with the module-trait correlation values and changes in expression patterns observed between TP1 and TP2 (Figure 6D).

Functional enrichment analysis identified different processes associated with the significantly correlated modules *AKNA*, *FYN*, *UBE2D3* and *UXS1* (Supplementary Table S9). Notably, the *AKNA* and *UBE2D3* modules were among the three modules significantly correlated with the musical stimuli after false discovery rate (FDR) correction (FDR < 0.05); Figure 6E. The *AKNA* module, which was downregulated in response to the stimuli, was primarily enriched for processes involving GTPase-mediated signal transduction, including RAS protein signaling pathways. Additionally, this module was also associated with immune-related functions such as myeloid cell differentiation and activation, B-cell differentiation and signaling, and the regulation of inflammatory responses, including interleukin-1 (IL-1) production (Figure 6E). On the other hand, only two enriched pathways were identified among the genes included in the upregulated *UBE2D3* module, namely “response to endoplasmic reticulum stress” and “cellular response to unfolded protein”. ORA analysis of the genes belonging *BCL10* module did not reveal any significantly enriched pathways.

In addition, we further explored the functional roles of the hub genes from the significantly correlated modules (*AKNA*, *UBE2D3* and *BCL10*) by retrieving the GO terms in which these genes are involved (Supplementary Table S10; Supplementary Figure S2). The *AKNA* gene is associated with 27 pathways, primarily related to nervous system processes (i.e. neurogenesis, forebrain development or stress response) as well as cell-cell adhesion. *UBE2D3* gene participates in 50 pathways, mainly linked to ubiquitination, protein-related catabolic processes and response to BMP and TGF-beta signaling. Finally, *BCL10* is involved in 157 pathways, predominantly associated with immune-related functions, cell–cell adhesion, and apoptotic processes.

### ASD-related genes vs. genes altered by musical stimuli

To explore the potential relevance of musical stimulation to autism-related molecular mechanisms, we compared the gene expression data from our merged dataset with the SFARI gene database. Of the 6,305 genes identified, 519 matched entries in the SFARI database. Among these, ten genes showed significant differential expression (*P*-value <0.05 and |Log_2_FC| > 1): nine were upregulated and one was downregulated. Notably, genes such as *EIF4E*, *DLGAP1*, and *CDKL5* were among the upregulated group, while *PTBP2* was significantly downregulated. Several of these genes have strong or suggestive associations with autism, according to their SFARI scores (e.g., *EIF4E*–Score 2; *CDKL5*-Score 1S). The identified genes are implicated in various processes, including synaptic signaling, protein translation, and RNA processing, suggesting that music stimulation may influence molecular pathways commonly affected in ASD (Supplementary Table S11).

### Exploratory analysis of salivary microbiota following musical stimulation

We also investigated whether musical stimulation induces detectable changes in the salivary microbiota by performing metagenomic inference on reads that failed to align to the human genome. As expected, a substantial proportion of these unmapped reads could not be matched to any bacterial reference genome (Supplementary Figure S3). This result is consistent with the complex and heterogeneous composition of saliva, which contains not only bacteria but also fungi, viruses, dietary RNA fragments, and environmental contaminants that may not be represented in the reference database used.

Applying a filtering criterion consistent with the human differential gene expression analysis (retaining only species with ≥10 reads in at least five samples), the number of species was reduced from 3,210 (Human-Enriched dataset) and 3,345 (Poly-A dataset) to 1,340 and 1,767 species, respectively. A total of 1,273 species were shared between both datasets.

**FIGURE 7:**
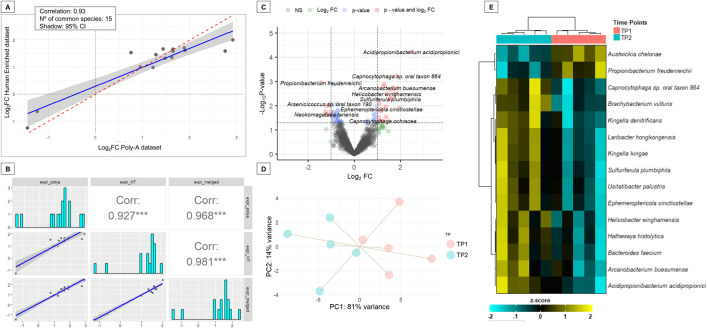
Differential abundance analysis of salivary microbial species pre- and post-musical stimulation in ASD subjects. **(A)** Scatter plot showing the Pearson correlation of Log_2_FC values for 15 microbial species with differential abundance (*P*-value <0.05) in both the Human-Enriched and Poly-A datasets. **(B)** Correlation between Log_2_FC values from the species from the intersection between differentially abundant species (*P*-value <0.05) from the merged dataset and the species with differential abundance previously found in common between the analysis of Human-Enriched and the Poly-A datasets, which were 15. **(C)** Volcano plot of the merged dataset displaying species with significant differential abundance. The plot shows the Log_2_FC on the x-axis and the -log_10_ *P*-value on the y-axis. Species with a *P*-value <0.05 and a |Log_2_FC| > 0.5 are colored in red, species with a *P*-value <0.05 but a |Log_2_FC| > 0.5 in light blue, species with an *P*-value >0.05 but a |Log_2_FC| > 0.5 in green and non-significant species in dark grey. **(D)** PCA plot based on abundance profiles from the merged dataset of the 15 commonly differentially abundant species. **(E)** Heatmap of the relative abundances from the merged dataset of the 15 commonly differentially abundant species.

Differential abundance analysis (DAA) was conducted using *DESeq2* in the two datasets separately. No bacterial species passed the adjusted significance threshold (adjusted *P*-value <0.05) in either dataset. However, 52 species in the Human-Enriched dataset and 62 in the Poly-A dataset showed nominal significance (*P*-value <0.05), with 15 species overlapping between the two datasets (Supplementary Table S12). A Pearson correlation analysis of the Log_2_FC for these 15 shared species revealed a high concordance (*r* = 0.93), indicating strong agreement between the independent analyses (Figure 7A).

Subsequently, a merged dataset was generated by aggregating read counts across datasets for the 1,273 shared species. This merged dataset showed also a high correlation in Log_2_FC values with the Poly-A dataset and Human-Enriched datasets for the 15 shared species between the two libraries (Figure 7B). DAA identified 68 species with significantly different abundances at TP2 compared to TP1 (*P*-value <0.05; Supplementary Table S13; Figure 7C). Notably, all 15 species previously found in common between the Human-Enriched and Poly-A datasets were also detected in the merged dataset. Changes in the abundance of these 15 species effectively distinguished the pre-stimulation and post-stimulation states in ASD subjects, as demonstrated by both PCA (Figure 7D) and heatmap analysis (Figure 7E). Unlike the separate analyses, this integrated approach identified one additional differentially abundant species, *Acidipropionibacterium acidipropionici*, with an adjusted *P*-value of 0.076 and a Log_2_FC of 2.5 (Supplementary Table S13).

## Discussion

Although RNA-seq is a powerful technique for biomarker discovery, saliva poses significant challenges due to its high bacterial RNA content and low abundance of human RNA, requiring specialized extraction and enrichment protocols (Ostheim et al., 2020; Gosch et al., 2024). In this study, we investigated the transcriptional effects of music exposure in the buccal mucosa of individuals with ASD using two complementary RNA-seq datasets prepared via Poly-A selection and Human-Enriched library protocols. Human-Enriched and Poly-A RNA-seq libraries exhibited distinct sequencing characteristics that align with their differing biological targets and preparation protocols. The aforementioned heterogeneous composition of saliva directly impacts the alignment metrics, resulting in lower mapping rates compared to the expected 70%–90% typically observed for RNA-seq reads from other sample types, such as blood (Dobin et al., 2013). Human-Enriched library showed higher duplication and GC content compared to Poly-A library preparation. Although it detected fewer genes, it achieved higher read counts per gene, reflecting greater sequencing depth for targeted transcripts (Conesa et al., 2016). This is expected, as Poly-A generally captures polyadenylated transcripts for an unbiased, transcriptome-wide analysis of coding genes, while Human-Enriched relies on hybrid capture with a targeted panel (e.g., the Exome Panel), limiting analysis to predefined genomic regions. Although each dataset individually revealed a poor mapping performance against the human genome and a modest number of DEGs, the integration of both datasets enabled the identification of a more consistent transcriptional signature, with enhanced robustness and biological interpretability. Reproducibility among technical replicates is generally expected to be high (Mortazavi et al., 2008). Despite the inherent technical differences between the Poly-A and Human-Enriched library preparation methods, our comparative analysis demonstrated a strong concordance in gene expression patterns between the two datasets. The moderate-to-high concordance in gene expression trends between the Poly-A and Human-Enriched datasets, and the high consistency in the merged dataset, reinforce the validity of the transcriptional changes observed and the integration strategy applied. Although the number of DEGs detected at stringent thresholds was limited, likely due to sample size constraints and biological variability inherent in human studies, the robustness of these shared signals underscores the value of integrating different RNA-seq strategies.

Three genes, *HERC6*, *TSPAN5*, and *REM2*, emerged as significant from the merged Poly-A selection and Human-Enriched datasets, suggesting they may play important roles in the brain’s response to musical stimuli in individuals with ASD. The upregulation of *HERC6* may point to a role for immune-related pathways in mediating the effects of music exposure. *HERC6* encodes an E3 ubiquitin-protein ligase and is a member of the small HERC family, which, unlike its larger counterparts (*HERC1*, *HERC2*), is thought to have evolved independently. While in many species *HERC6* participates in type I interferon responses by facilitating ISGylation, in humans ISGylation activity is mediated by *HERC5* (Sanchez-Tena et al., 2016). However, emerging evidence suggests that *HERC6* may still play roles in antiviral defense and immune regulation through alternative, non-ISGylation-based mechanisms (Oudshoorn et al., 2012). The modulation of such immune-related genes is particularly relevant given accumulating evidence that immune dysregulation is a common feature in ASD. Music, by influencing stress levels and immune signaling pathways, could indirectly affect the local expression of genes such as *HERC6*, potentially contributing to homeostatic recalibration in neuroimmune interactions. Perhaps more directly tied to neurodevelopmental processes, the upregulation of *TSPAN5* presents a compelling finding. *TSPAN5* encodes a glycoprotein of the tetraspanin family, which is involved in a broad array of cellular functions including adhesion, migration, and proliferation. Of particular importance is its role in the central nervous system, where it is highly expressed in the forebrain and cerebellum. Recent studies have demonstrated that *TSPAN5* facilitates the maturation of dendritic spines by promoting the synaptic clustering of neuroligin-1 (NLG-1) (Moretto et al., 2019), a protein that has been closely linked to ASD-related social behavior deficits (Rawsthorne et al., 2021). The upregulation of *TSPAN5* in the buccal mucosa of ASD patients following musical intervention may thus reflect a shift toward synaptic stabilization and maturation occurring in the brain. This aligns with previous reports indicating that music-based therapies can improve cognitive and social functioning in individuals with ASD, potentially through activity-dependent plasticity and enhanced synaptic integration. Conversely, *REM2* was significantly downregulated after music exposure. *REM2* belongs to the RGK subfamily of Ras-like GTPases and plays a critical role in neuronal differentiation and synaptic development. However, intriguingly, inhibition of *REM2* has also been associated with increased dendritic branching and arborization, resulting in more structurally complex neurons (Ghiretti and Paradis, 2011). This dualistic effect complicates the interpretation of its downregulation, yet suggests that reduced *REM2* expression may contribute to neuroplastic adaptations rather than dysfunction *per se*. In the context of this study, the simultaneous upregulation of *TSPAN5* may offset potential drawbacks of *REM2* suppression, together fostering a structural environment conducive to improved neural integration and function.

The concurrent modulation of *HERC6*, *TSPAN5*, and *REM2* in the buccal cavity of ASD individuals after music exposure appear to span multiple functional domains, including immune regulation, synaptic maturation, and structural remodeling, all of which are relevant to the ASD phenotype. While the directionality and interplay between these pathways remain to be fully elucidated, our findings resonate with a growing body of literature suggesting that music can serve as a multisensory and emotionally salient stimulus capable of driving neuroplasticity.

Numerous studies have explored the relationship between ASD and alterations in the synaptic excitation-inhibition (E/I) ratio. Some have reported an increased E/I ratio in individuals with ASD (Rubenstein and Merzenich, 2003), while others, particularly in Down syndrome, have suggested that heightened inhibition may be the primary contributor to learning deficits (Belichenko et al., 2009). Collectively, these findings seem to indicate that homeostatic regulation of neural activity is often disrupted in ASD. Given the heterogeneity of the disorder, it is plausible that both hyperexcitation and hyperinhibition could give rise to distinct ASD subtypes or manifestations. GSVA revealed a coordinated downregulation of neurodevelopmental and synaptic pathways in the buccal mucosa of ASD patients after music exposure, contrasting with the global upregulation pattern observed across all significant pathways. We hypothesize that the observed downregulation of most neural DRPs following musical stimuli may represent a homeostatic response aimed at modulating excessive synaptic excitation, a characteristic frequently observed in ASD. This response could reflect a reduction in the E/I ratio, underscoring the potential of music as a modulator of neural excitability. The cellular response to corticosteroid stimulus was the only significantly upregulated pathway, which may represent a stress-related or neuroendocrine response to the stimulus. Corticosteroids are hormones involved in stress regulation and immune modulation, mainly through the hypothalamic–pituitary–adrenal axis (HPA) (Agorastos et al., 2019; Wong et al., 2022). HPA axis dysregulation is a well-documented feature of ASD (Spratt et al., 2012). HPA activity is correlated with cortisol levels, an indicator of stress. ASD often exhibits atypical cortisol secretion patterns and blunted or exaggerated stress reactivity (Spratt et al., 2012; Taylor and Corbett, 2014). Our data suggest that music exposure may transiently enhance corticosteroid-responsive gene expression, reflecting a temporary adjustment of stress-adaptive mechanisms rather than a stress response in these individuals. Further studies are required to clarify the functional significance of this pathway activation and its potential role in mediating the effects of music in ASD. In contrast, our ORA revealed significant enrichment in pathways associated with immune processes, particularly those involving the proliferation and differentiation of immune cells such as leukocytes, lymphocytes, and mononuclear cells. Immune dysregulation is a well-established co-occurring condition in ASD, with several studies reporting elevated levels of TNF-α, IL-6, granulocyte colony-stimulating factor (G-CSF), IFN-γ, and IL-8 in the brain, along with a heightened pro-inflammatory Th1 cytokine profile (Li et al., 2009; Wei et al., 2011; Robinson-Agramonte et al., 2022). Music exposure modulates immune-related pathways, suggesting an attenuation of pro-inflammatory states, supporting previous findings that music can exert immunoregulatory effects and stress-related responses.

While further mechanistic studies are warranted, our GSVA and ORA results support the notion that sensory stimuli such as music may trigger compensatory transcriptional programs in individuals with ASD, influencing neural excitability and immune function. Cross-referencing music-responsive DEGs with the SFARI gene database revealed modulation of genes involved in synaptic structure, intracellular signaling, and RNA metabolism, pathways commonly disrupted in ASD. Notably, several upregulated genes, including *DLGAP1* and *CDKL5*, play critical roles in synaptic scaffolding and neuronal morphogenesis (Chen et al., 2010; Shin et al., 2012). *DLGAP1* encodes a postsynaptic protein localized at glutamatergic synapses, while *CDKL5* is essential for the stability of excitatory synapses (Chen et al., 2010). The altered expression of these genes suggests a potential link between musical stimulation and enhanced synaptic plasticity in ASD-related contexts. In addition, genes such as *EIF4E*, *STYK1*, and *CDKL5* are components of the PI3K/Akt/mTOR signaling pathway, a key regulator of synaptic protein synthesis and neural development. Importantly, we also observed the downregulation of *PTBP2*, a central regulator of neuronal exon splicing and RNA stability. Along with other RNA-binding genes such as *UPF3B* and *FRG1*, this finding indicates that music may exert broader effects on RNA processing and transcript diversity (Sun et al., 2011; Maracci et al., 2022). Taken together, these transcriptional changes suggest that music stimulation can reprogram gene expression patterns across interconnected molecular pathways, potentially contributing to neuroplastic and compensatory mechanisms relevant to ASD.

We identified different co-expression modules whose activity patterns were significantly correlated with exposure to musical stimuli in ASD donors. This suggests that musical stimuli may engage specific regulatory networks in the buccal mucosa, potentially reflecting broader systemic or brain-related transcriptional responses. From the three main significantly correlated modules, *BCL10* and *UBE2D3* showed a global upregulation pattern in response to music stimulation, while the *AKNA* module demonstrated an overall downregulation. Notably, hub gene *AKNA* has already been previously reported as a novel ASD risk gene, carrying *de novo* variants probably related to ASD (Kim et al., 2020). *AKNA* module was functionally enriched for pathways related to GTPase-mediated signal transduction, with a particular emphasis on Ras protein signaling, suggesting that musical stimulation affects specific intracellular signaling cascades related to these small GTPases. The Rho family of GTPases (members of the larger Ras superfamily) are small signaling proteins that play pivotal roles in neural development, including axon guidance, dendritic spine formation, and synaptic plasticity processes (Zhang et al., 2021). Importantly, dysregulation of Rho GTPase signaling has been increasingly recognized as a contributing factor in various neuropathological events, including ASD (Arrazola Sastre et al., 2020; Guo et al., 2020). Multiple lines of evidence have highlighted aberrant activity of Rho family members, such as Rac1, in ASD-related phenotypes both in human studies and animal models (Zeidan-Chulia et al., 2016). Interestingly, the hub gene of this module (*AKNA*) is directly involved in molecular pathways such as neurogenesis or forebrain development, indicating a potential regulatory role of this module in nervous system-related processes. In addition, this module was also enriched for genes involved in immune-related processes. The immunomodulatory effect of the musical stimuli appears to act through the suppression specific immune cells populations. The downregulation of inflammatory and IL-1 pathways could be of particular interest given the role of neuroinflammation in the pathophysiology of ASD (Ricci et al., 2013; Careaga et al., 2017; Saghazadeh et al., 2019). Module analysis also revealed a selective activation of stress-response mechanisms (via the *UBE2D3* module). The accumulation of unfolded proteins in the endoplasmic reticulum (ER) triggers ER stress, specifically activating a protective unfolded protein response in the ER. Several studies have identified ER stress as a significant contributor to the pathophysiology of ASD (Fujita et al., 2010). ER stress has also been shown to impair neurite outgrowth, alter synaptic protein expression, and disrupt neuronal differentiation, all of which are relevant to ASD neurodevelopmental deficits (Kawada and Mimori, 2018). In addition, upregulation of ER stress-related genes has been reported in the brains of ASD individuals (Crider et al., 2017; Kawada and Mimori, 2018). Our results indicate that music exposure may enhance ER stress response mechanisms in buccal cells, potentially regulated by genes within *UBE2D3* module, which could help to compensate for the impact of stress pathways implicated in ASD.

While the metagenomic analysis conducted in this study was exploratory and limited in scope, it yielded several notable observations. The large fraction of sequencing reads that failed to align with bacterial reference genomes highlights the inherent complexity of salivary metagenomic samples. Saliva is a biologically diverse medium that includes bacteria, fungi, viruses, dietary residues, and environmental RNA fragments, many of which are absent from current bacterial reference databases. Dysbiosis of the oral microbiome has previously been linked to ASD, potentially contributing to neurodevelopmental alterations via the gut–brain axis or immune modulation (Wong et al., 2021; D'Angelo et al., 2025). A subset of taxa showed consistent nominal differences between pre- and post-stimulation samples across both datasets. In particular, 15 species demonstrated shared differential abundance patterns with high correlation in Log_2_FC (r = 0.93), suggesting reproducibility across sequencing approaches (Human-Enriched vs. Poly-A datasets). Notably, the merged dataset identified *Acidipropionibacterium acidipropionici* as a potentially modulated species following musical stimulation (adjusted *P*-value = 0.076). This acid-producing bacterium is known for its role in dairy fermentation (Coronas et al., 2023), and closely related propionibacteria have been previously identified as part of the oral microbiota (Sato-Suzuki et al., 2020). We also identified *Propionibacterium freudenreichii*, a close relative involved in propionic acid production, among our top 15 candidate species. There is substantial evidence implicating propionibacteria in neurological effects via propionic acid production, an important short-chain fatty acid. Elevated propionic acid levels in the gut have been associated with ASD-like behaviors and neuroinflammation in animal models (Srikantha and Mohajeri, 2019). According to Li and Zhou (2016), 3-(3-hydroxyphenyl)-3-hydroxypropionic acid induces autism symptoms by emptying and depleting catecholamines in the brain. El-Ansary et al. (2012), El-Ansary et al. (2015) reported that the neurotoxicity of propionic acid could play a central role in the etiology of autistic biochemical features. This finding raises the intriguing possibility that auditory stimulation may exert an “echo effect” on the composition of the oral microbiome. These results invite further inquiry into the interplay between sensory stimuli and microbial communities. It remains unclear whether the observed microbial shifts are directly mediated by neural or physiological responses to music, or indirectly through changes in salivary secretion, flow rate, or composition. Future metagenomic studies with larger cohorts, increased sequencing depth, and broader reference annotations, including non-bacterial taxa, are needed to validate and expand on these preliminary findings. Further research should explore the potential of targeting the microbiota–gut–brain axis through musical stimuli as a therapeutic strategy in ASD, in parallel with approaches such as probiotic supplementation (Li and Zhou, 2016; Feng et al., 2023), or more broadly, interventions that manipulate the enteric microbiome to alleviate autism symptoms (Frye et al., 2015).

One of the most significant challenges encountered in the present study was the use of saliva as the biological matrix for transcriptomic analysis. While saliva sampling offers clear advantages, being non-invasive, stress-free, and highly accessible, particularly in sensitive populations such as individuals with ASD, it also presents substantial technical hurdles. Saliva contains a heterogeneous mix of host epithelial cells, immune cells, and a rich microbial community, which can result in low yields of high-quality human RNA and high proportions of unmapped or non-human reads. These factors complicate both the quality control and the interpretation of gene expression data. Despite these challenges, this study demonstrates that with careful preprocessing, rigorous quality filtering, and thoughtful experimental design, it is possible to extract biologically meaningful transcriptomic signals from saliva. This opens the door for future research into peripheral biomarkers of brain function and neuroplasticity in contexts where blood collection is difficult or ethically sensitive, such as pediatric or neurodiverse populations. To further advance the utility of saliva transcriptomics, future studies should aim to improve RNA stabilization protocols, integrate host-microbiome transcriptome analyses, and consider single-cell or spatial transcriptomics to better resolve cell-type-specific expression patterns. Combining saliva-based transcriptomics with behavioral, neuroimaging, or immune profiling could yield a more comprehensive understanding of how systemic biological pathways contribute to neurodevelopmental conditions like ASD.

This study is not without limitations. The sample size, while reasonable for a pilot analysis, limits the power to detect subtle effects and may also influence the stability of enrichment analyses, whose robustness depends on the reliability of the input gene list. Nonetheless, the enrichment of pathways consistently associated with ASD, such as immune regulation and mitochondrial function, supports the biological relevance of our findings. The exclusion of outlier samples was necessary to reduce noise but may have inadvertently excluded biologically informative variance. Additionally, the focus on peripheral tissue (saliva) restricts direct extrapolation to brain-specific mechanisms, although saliva has increasingly been recognized as a viable proxy for systemic and neuroimmune interactions. Future studies should build upon these findings by increasing cohort sizes, incorporating longitudinal designs, and using complementary modalities such as neuroimaging and behavioral assessments. Furthermore, investigating cell-type-specific transcriptomic responses or incorporating single-cell RNA-seq could help disentangle the contributions of diverse cell populations and improve the resolution of observed effects.

In conclusion, this study offers preliminary yet compelling evidence that music exposure can modulate the expression of genes involved in synaptic plasticity, neuronal architecture, and immune signaling in individuals with ASD. Notably, the upregulation of *TSPAN5* and *HERC6*, alongside the downregulation of *REM2*, suggests a transcriptional profile that may favor enhanced neural adaptability and integration. These molecular findings enrich our understanding of the biological mechanisms through which music may exert therapeutic effects and point to promising avenues for developing targeted, non-invasive interventions in neurodevelopmental conditions. Furthermore, given the frequent reports of oral microbiota dysbiosis in ASD, the observed trends in microbial composition following musical stimulation, though exploratory, raise important questions about the broader systemic effects of sensory inputs. Understanding how music may influence the oral microbiome could open new perspectives on modulating host–microbe interactions through behavioral or environmental stimuli (Castelo-Martínez et al., 2025). Nevertheless, these initial findings should be interpreted with caution, and future research with larger cohorts and expanded metagenomic approaches will be essential to confirm and contextualize these effects.

## Data Availability

The raw count data from the human-merged and the metagenomic-merged datasets, along with the metadata, are publicly available in the Figshare repository under the accession: https://doi.org/10.6084/m9.figshare.29504588.

## Supplementary material

SUPPLEMENTARY FIGURE S1
**(A)** Plots showing correlation between soft-thresholding powers and both the scale-free fit index and the mean connectivity (upper). **(B)** Dendrogram of genes and co-expression modules detected, represented by different colors.

SUPPLEMENTARY FIGURE S2 Clustering of the semantic similarity matrix obtained from the Gene Ontology (GO; subcategory biological processes) terms in which hub genes from the significantly correlated modules are involved.

SUPPLEMENTARY FIGURE S3 Pie charts representing the percentage of bacterial reads and unknown reads in the Human-Enriched dataset **(A)** and in the Poly-A dataset **(B)**.
