# Supplementary figures and images for: Decoding the peripheral transcriptomic and meta-genomic response to music in autism spectrum disorder via saliva-based RNA sequencing
Source: Front Mol Biosci. 2025 Nov 24;12:1696704. doi: 10.3389/fmolb.2025.1696704 (PMC12682756; doi:10.3389/fmolb.2025.1696704)

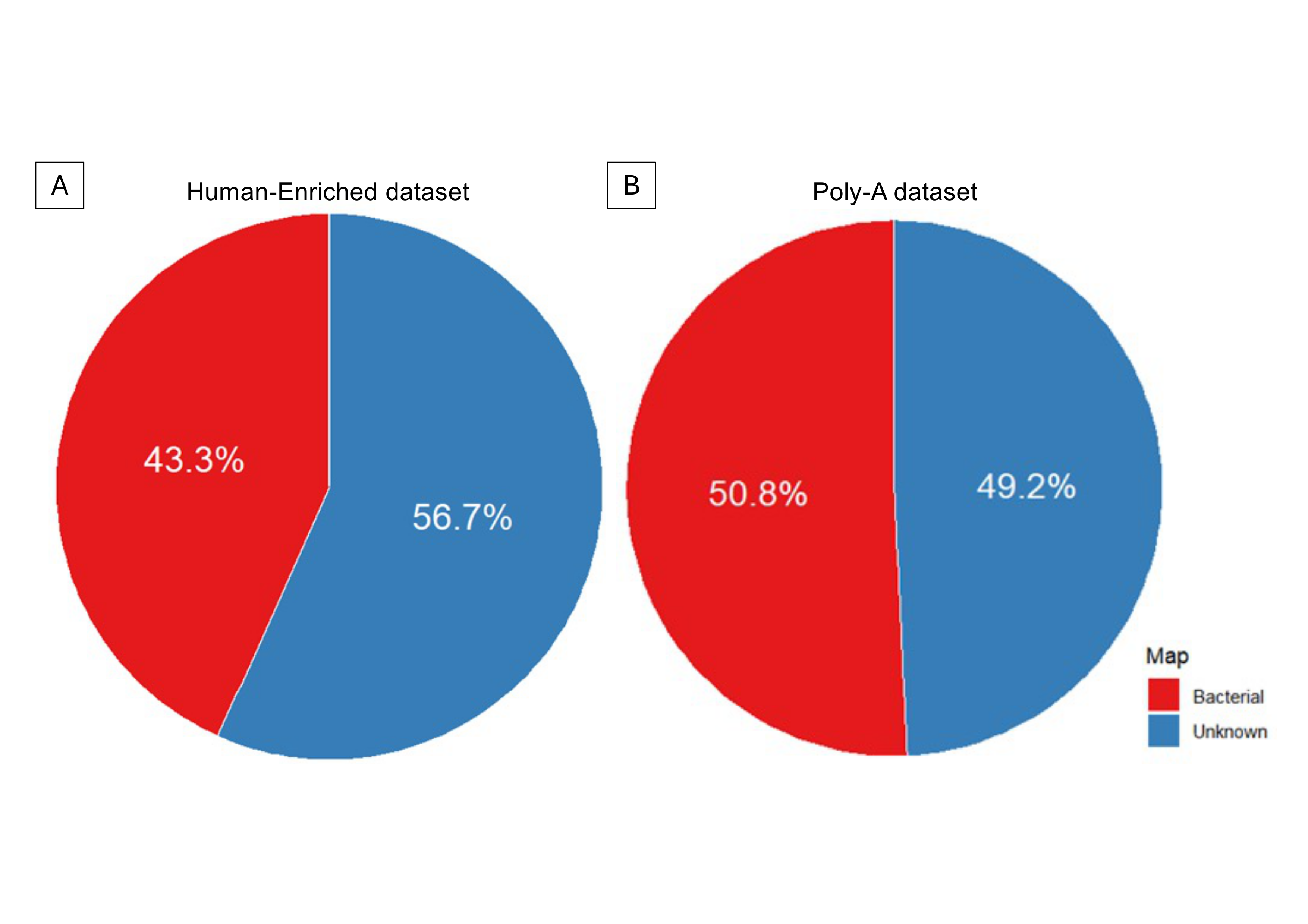

Supplement: Supplementary file 5 [file Image3.tif]

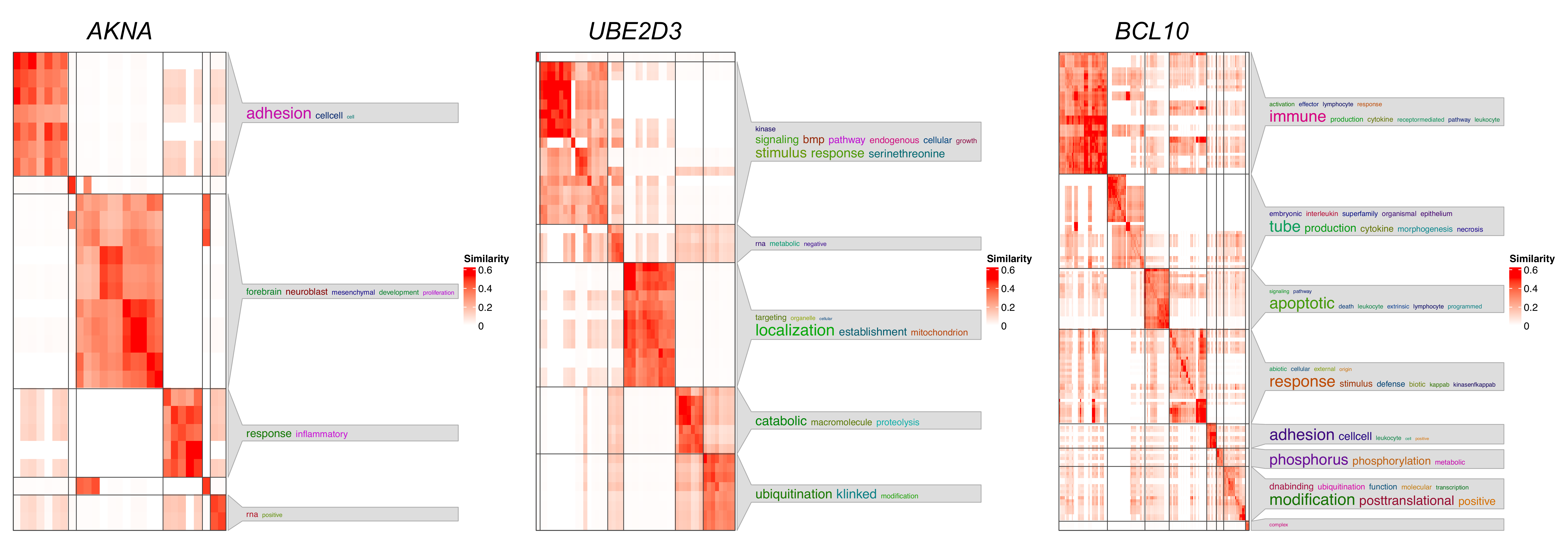

Supplement: Supplementary file 6 [file Image2.tif]

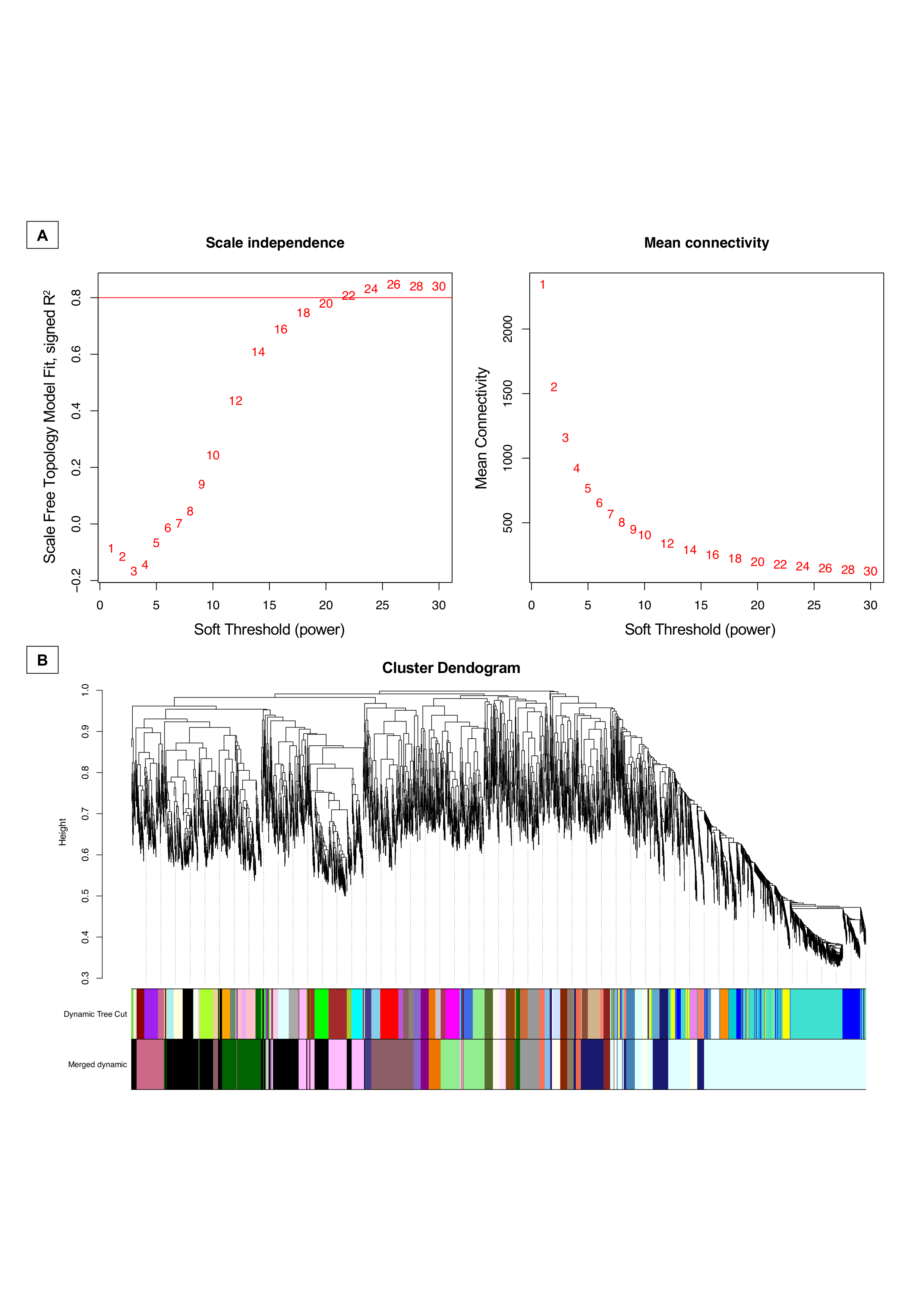

Supplement: Supplementary file 7 [file Image1.tif]
